# Age adjustment in ecological studies: using a study on arsenic ingestion and bladder cancer as an example

**DOI:** 10.1186/1471-2458-11-820

**Published:** 2011-10-20

**Authors:** How-Ran Guo

**Affiliations:** 1 Department of Environmental and Occupational Health, College of Medicine, National Cheng Kung University, Tainan, Taiwan; 2 Department of Occupational and Environmental Medicine, National Cheng Kung University Hospital, Tainan, Taiwan; 3 Sustainable Environment Research Center, National Cheng Kung University, Tainan, Taiwan

**Keywords:** ecological study, age adjustment, direct standardization, indirect standardization, standardized morbidity ratio, arsenic, drinking water, bladder cancer

## Abstract

**Background:**

Despite its limitations, ecological study design is widely applied in epidemiology. In most cases, adjustment for age is necessary, but different methods may lead to different conclusions. To compare three methods of age adjustment, a study on the associations between arsenic in drinking water and incidence of bladder cancer in 243 townships in Taiwan was used as an example.

**Methods:**

A total of 3068 cases of bladder cancer, including 2276 men and 792 women, were identified during a ten-year study period in the study townships. Three methods were applied to analyze the same data set on the ten-year study period. The first (Direct Method) applied direct standardization to obtain standardized incidence rate and then used it as the dependent variable in the regression analysis. The second (Indirect Method) applied indirect standardization to obtain standardized incidence ratio and then used it as the dependent variable in the regression analysis instead. The third (Variable Method) used proportions of residents in different age groups as a part of the independent variables in the multiple regression models.

**Results:**

All three methods showed a statistically significant positive association between arsenic exposure above 0.64 mg/L and incidence of bladder cancer in men and women, but different results were observed for the other exposure categories. In addition, the risk estimates obtained by different methods for the same exposure category were all different.

**Conclusions:**

Using an empirical example, the current study confirmed the argument made by other researchers previously that whereas the three different methods of age adjustment may lead to different conclusions, only the third approach can obtain unbiased estimates of the risks. The third method can also generate estimates of the risk associated with each age group, but the other two are unable to evaluate the effects of age directly.

## Background

In spite of their limitations, ecologic studies are often used in epidemiologic research. These studies gather study participants into groups, mostly according to the geographic area of residence, and treat the whole group of people as a unit [1]. In ecological studies, distribution of age in the unit population may affect the results, and therefore evaluating and adjusting for age effects are desirable in many cases. In regression analyses, a common approach is to apply direct standardization method [2] to obtain the age-standardized risk of each unit population and then use it as the dependent variable. Another common approach, especially frequently applied in the SMR ("standardized morbidity ratio" or "standardized mortality ratio") studies, is to adopt indirect standardization method to obtain age-standardized risk ratio [3] of each unit population and then use it as the dependent variable instead. The third technique is to treat age as a predictor and adjust for its effects by adding independent variables [4,5].

Different approaches, however, may lead to different results. Rosenbaum and Rubin [4]have shown that the third approach can generate unbiased risk estimates as those obtained from simple linear regression models using data on individual participants. They also showed that the first approach generally cannot generate unbiased risk estimates and suggested avoiding its use except when the bias can be shown to be negligible for the purposes of the study. However, they did not provide any empirical examples in the paper. Likewise, the second approach has been recognized as one of the "common errors in disease mapping" by Ocaña-Riola [6], but no empirical examples were included in the paper. Therefore, the objective of this study is to compare these three approaches using an ecologic study on the associations between arsenic levels in drinking water and the incidence of bladder cancer in Taiwan as an empirical example. On the basis of the three approaches, three regression models were applied to model the relations between each of the outcome measurements (age-adjusted mortality rate, standardized mortality rate, and crude mortality rate) and exposure levels.

The association between consumption of artesian well water and cancer in Taiwan has been documented since the 1960s [7-18], and bladder cancers had the highest relative risks [13,19]. All the three methods have been used to study the associations between arsenic ingestion and cancer in Taiwan [7-9], but most previous studies were limited to 6 townships in the southwestern coast area. The current study evaluated the associations between arsenic ingestion and bladder cancer using data on 243 townships in Taiwan, including the 6 most frequently studied.

## Methods

### Arsenic levels in drinking water

Data on the arsenic levels in drinking water were obtained from a nation-wide survey conducted by the Taiwan Provincial Institute of Environmental Sanitation [20] using the standard mercuric bromide stain method [21]. According to the standard solutions used in that survey, drinking water arsenic levels can be grouped into 10 categories: "undetectable" (test result compatible with the blank control) "trace" (test result between the blank control and the 0.01 mg/L standard), "0.01 mg/L," "0.02 mg/L," "0.03-0.04 mg/L," "0.05-0.08 mg/L," "0.09-0.16 mg/L," "0.17-0.32 mg/L," "0.33-0.64 mg/L," and " > 0.64 mg/L." While Taiwanese laboratories applied this method, the limit of detection (LOD; defined as the value of the mean plus three times of the standard deviation obtained from repetitive testing of blank controls) was 0.04 mg/L [22]. Therefore, in the data analyses, all levels at or below the LOD were combined together as a single category " < 0.05 mg/L."

The original survey data are available for 65269 wells in 243 townships, with an average of about 269 wells in each township. Because the survey was specifically for the arsenic level in drinking water, the standard report form did not include other chemical characteristics of the well water. As in most of the similar ecological studies, the number of users of each well was not recorded. The survey performed almost all the measurements between 1974 and 1976. At the time of survey bottled water was generally unavailable, and therefore it can be assumed that most quantity of drinking water was taken from the same source all days in the surveyed areas.

Data on the number of residents by gender and age in each township were obtained from the Department of Internal Affairs to estimate the number of people in each unit population. The 243 townships had a total of about 11.7 million residents, about half of the population in Taiwan, and Table 1 presents the estimated number of people in each exposure category. About 48000 people drank water in the highest arsenic category (" > 0.64 mg/L"), which was the smallest population in an exposure category.

**Table 1:** Distribution of arsenic exposure levels in well water

	Arsenic level (mg/L)					
	< 0.04	0.05-0.08	0.09-0.16	0.17-0.32	0.33-0.64	> 0.64
Average % of wells in each township	91.0	2.8	3.0	1.7	1.0	0.6
Estimated population^a^						
Males	5 605 000	169 000	159 000	84 000	47 000	25 000
Females	5 144 000	156 000	147 000	77 000	43 000	23 000
Total	10 749 000	326 000	305 000	162 000	90 000	48 000

^a^round-off to 1000; estimated total study population: 11 678 000.

### Collection of other data

Cases of bladder cancer diagnosed between January 1, 1980 and December 31, 1989 were identified using the computerized database of the National Cancer Registry Program, which is operated by the Department of Health. Gender, age, diagnoses, and township of residence were reported for each registered case. Cases with ICD-O codes [23] from 188.0 to 188.9 were defined as bladder cancer cases, and 3068 cases, including 2276 men and 792 women, were identified.

Demographic data on the residents in each township at the end of 1985, the midpoint of the ten-year study period, were obtained from the Department of Internal Affairs. The numbers of residents of seven age groups were calculated: 0-19 years, 20-29 years, 30-39 years, 40-49 years, 50-59 years, 60-69 years, and above 69 years.

An urbanization index developed by Wu [24] on the basis of 19 socioeconomic factors was adopted to assess the associations between urbanization and incidence of bladder cancer. The study townships had urbanization indexes ranging from -1.410 to 3.257 (mean = 0.224, standard deviation = 1.128).

The magnitude of cigarette sales was used to evaluate effects of smoking. In Taiwan, cigarette selling was a monopoly business operated by the Tobacco and Alcohol Monopoly Bureau during the study period. Sales records collected from the Bureau in a previous study [7] were adopted to estimate the number of cigarettes sold per capita per year in each township, which had a range of 14.94 to 689.93 (mean = 63.76, standard deviation = 66.11). The unit for of cigarette sales used in the analyses was 100 cigarettes.

### Data analysis

For comparison, three different methods were applied to analyze the data, but they required different information. To account for the fact that the size of the population was different across the townships, in all three approaches, the population in each township was used as the weighting factor in regression models.

The first approach, referred to as "Direct Method," applies the direct standardization procedure. For each township, gender-age specific cumulative incidence rates over the ten-year period were calculated, and then a standardized incidence rate (SIRate) can be obtained by adopting the age distribution of the world standard population in 1976 [25] as the following:

SIRate=Σ(Wi×IRi)∕ΣWi,

where W*i *is the number of people in the *i*th age group in the standard population, and IR*i *is the age-specific average annual cumulative incidence rate of the *i*th age group. The unit for IR*i *was cases per 100,000. Then, the risk associated with each exposure level can be estimated through the following regression model:

(1) $$\text{SIRate}=\alpha +\beta_{1}{\text{X}}_{1}+...+\beta_{5}{\text{X}}_{5}+\gamma \text{U}+\delta \text{T}$$

where for each township, X*j *is the proportion (as percentage) of residents with arsenic exposures in category *j*, U is the urbanization index, and T is the number of cigarettes (in hundreds) sold per capita. Because the exposure category " < 0.05 mg/L" was used as the reference, X_1 _= percentage of residents in the "0.05-0.08 mg/L" category, X_2 _= percentage of residents in the "0.09-0.16 mg/L" category, and so on. In this case, α (intercept) is the estimated background cumulative incidence rate, β*j *indicates the rate difference (RD) associated with each 1% increase in residents in category *j*, γ indicates the RD associated with each one-unit increase in urbanization index, and δ indicates the RD associated with each 100 cigarettes sold per capita.

The second approach, referred to as "Indirect Method," applies the indirect standardization procedure, which adopts the age-specific incidence rates in a reference population and obtains the expected number of cases in the *i*th age group (E*i*) in a given township as the following:

Ei=Pi×RIRi,

where P*i *is the number of people in the *i*th age group in the township, and RIR*i *is the age-specific cumulative incidence rate of the *i*th age group in the reference population. The total population of the 243 townships combined was used as the reference population in the analysis. A standardized incidence ratio (SIRatio) for each township can thus be obtained as the following:

SIRatio=ΣOi∕ΣEi,

where O*i *is the observed number of cases in the *i*th age group in the unit population. Then, the risk associated with each exposure level can be estimated through the following regression model:

(2) $$\text{SIRatio}=\alpha^{\prime }+{\beta_{1}}^{\prime }{\text{X}}_{1}+...+{\beta_{5}}^{\prime }{\text{X}}_{5}+\gamma^{\prime }\text{U}+\delta^{\prime }\text{T}$$

where X*j*, U, and T are defined as in Model 1. In this case, α' is the estimated background ratio, β*j*' indicates the increase in SIRatio associated with each 1% increase in residents in category *j*, γ' indicates the increase in SIRatio associated with each one-unit increase in urbanization index, and δ' indicates the increase in SIRatio associated with each 100 cigarettes sold per capita. In this model, SIRatio (a rate ratio) needs to be forced to take the value 1 when the arsenic exposure is within the reference category (" < 0.05 mg/L") and all other variables are set to their reference categories. This can be accomplished through coding the " < 0.05 mg/L" group as the reference category.

The third approach, referred to as "Variable Method," treats age as a predictor of bladder cancer and adds independent variables in the regression models to evaluate and adjust for the effects of age as the following:

(3) $$\text{CIR}=\alpha^{\prime \prime }+{\beta_{1}}^{\prime \prime }{\text{X}}_{1}+...+{\beta_{5}}^{\prime \prime }{\text{X}}_{5}+\gamma^{\prime \prime }\text{U}+\delta^{\prime \prime }\text{T}+\theta_{1}{\text{A}}_{1}+...+\theta_{6}{\text{A}}_{6}$$

where CIR is the crude cumulative incidence rate, X*j*, U, and T are defined as in Model 1, and A*k *is the proportion (as percentage) of residents in age group *k *in each township. Because there are seven age groups, six independent variables derived from dummy variables at the individual level were used in the regression model [5]. Therefore, the age group "0-19 years" was used as the reference, A_1 _= percentage of residents in the age group "20-29 years," A_2 _= percentage of residents in the age group "30-39 years," and so on. In this case, α" is the estimated background cumulative incidence rate; β*j*", γ", and δ" are defined as β*j*, γ, and δ in Model 1 respectively; and θ*k *indicates the RD associated with each 1% increase in residents in age group *k*.

Models 1 and 3 generate estimates of RD's, but Model 2 generates estimates of incremental rate ratios. Therefore, estimates of RD's from Models 1 and 3 were then divided by the estimates of background rates (α and α" respectively) to obtain estimates of incremental rate ratios to facilitate the comparison among the three methods.

## Results

For men, all three methods showed a statistically significant positive association between arsenic exposure above 0.64 mg/L and incidence of bladder cancer, but not for the other exposure categories. (Table 2) However, the risk estimates obtained by different methods for the same exposure category were all different. For women, the dose-response relationships appeared to be irregular. (Table 3) All three methods showed a significant positive association between arsenic exposure above 0.64 mg/L and incidence of bladder cancer as well as a significant negative association with bladder cancer incidence for arsenic exposures between 0.09 and 0.16 mg/L. All three methods showed a negative association with bladder cancer incidence for arsenic exposure between 0.16 and 0.32 mg/L, but when the Variable Method was used, the association would be determined as not significant by the general practice of setting the significant level at 0.05 (p = 0.08). Similarly, the risk estimates obtained by different methods for the same exposure category were all different. For both genders, the risk estimates obtained by the Indirect Method were between those by the Direct Method and those by the Variable Method for all exposure categories.

**Table 2:** Estimates of incremental relative risks (IRRs) for bladder cancer of men obtained by three different methods

Predictors	Direct Method	Indirect Method	Variable Method
	IRR [SE]	IRR [SE]	IRR [SE]
Arsenic Exposure^a^			
0.05-0.08 mg/L	-0.029 [0.021]	-0.023 [0.017]	-0.016 [0.010]
0.09-0.16 mg/L	0.056 [0.030]	0.042 [0.024]	0.022 [0.013]
0.17-0.32 mg/L	-0.054 [0.039]	-0.031 [0.032]	-0.012 [0.017]
0.33-0.64 mg/L	0.035 [0.045]	0.021 [0.036]	0.007 [0.020]
> 0.64 mg/L	0.274 [0.036]	0.228 [0.029]	0.115 [0.016]
Urbanization Index^b^	0.167 [0.046]	0.134 [0.037]	0.169 [0.040]
Cigarette Sale^c^	-0.047 [0.104]	-0.026 [0.084]	-0.006 [0.046]
Age^d^			
20-29 years	NA^e^	NA	0.021 [0.019]
30-39 years	NA	NA	-0.050 [0.026]
40-49 years	NA	NA	-0.014 [0.035]
50-59 years	NA	NA	-0.041 [0.028]
60-69 years	NA	NA	0.014 [0.028]
> 69 years	NA	NA	0.037 [0.040]
p Value for the Model^f^	< 0.001	< 0.001	< 0.001

^a^incremental relative risk for each 1% increase in residents exposed to arsenic levels in each exposure category.

^b^incremental relative risk for each one-unit increase in urbanization index.

^c^incremental relative risk for each 100 cigarettes sold per year.

^d^incremental relative risk for each 1% increase in residents in each age group.

^e^not included in the analyses.

^f^p value for F test of the significance of the model.

**Table 3:** Estimates of incremental relative risks (IRR) for women obtained by three different methods

Predictors	Direct Method	Indirect Method	Variable Method
	IRR [SE]	IRR [SE]	IRR [SE]
Arsenic Exposure^a^			
0.05-0.08 mg/L	-0.125 [0.045]	-0.077 [0.030]	-0.095 [0.046]
0.09-0.16 mg/L	0.259 [0.062]	0.162 [0.041]	0.214 [0.063]
0.17-0.32 mg/L	-0.222 [0.082]	-0.136 [0.055]	-0.143 [0.082]
0.33-0.64 mg/L	0.103 [0.094]	0.060 [0.062]	0.032 [0.093]
> 0.64 mg/L	0.489 [0.075]	0.349 [0.050]	0.354 [0.076]
Urbanization Index^b^	0.324 [0.097]	0.241 [0.064]	0.299 [0.212]
Cigarette Sale^c^	0.011 [0.219]	0.009 [0.146]	-0.004 [0.219]
Age^d^			
20-29 years	NA^e^	NA	0.122 [0.153]
30-39 years	NA	NA	-0.249 [0.146]
40-49 years	NA	NA	0.346 [0.217]
50-59 years	NA	NA	-0.170 [0.287]
60-69 years	NA	NA	0.065 [0.373]
> 69 years	NA	NA	-0.417 [0.304]
p Value for the Model^f^	< 0.001	< 0.001	< 0.001

^a^incremental relative risk for each 1% increase in residents exposed to arsenic levels in each exposure category.

^b^incremental relative risk for each one-unit increase in urbanization index.

^c^incremental relative risk for each 100 cigarettes sold per year.

^d^incremental relative risk for each 1% increase in residents in each age group.

^e^not included in the analyses.

^f^p value for F test of the significance of the model.

All three methods showed a significant positive association between urbanization index and incidence of bladder cancer for men, but the Indirect Method gave a lower risk estimate than the other two. (Table 2) Again, although all three methods showed a positive association between urbanization index and incidence of bladder cancer for women, when the Variable Method was used, the association would be determined as not significant by the general practice of setting the significant level at 0.05 (p = 0.16). (Table 3)

For both men and women, no significant association was found between cigarette sales and incidence of bladder cancer by any of the methods. (Tables 2 and 3) The risk estimates obtained by different methods were quite different, and that obtained by the Indirect Method was between those obtained by the Direct and the Variable Methods for both genders.

Among the three methods, only the Variable Method can generate risk estimates associated with different age groups. None of the six age groups had a significant higher risk. (Tables 2 and 3)

To determine the validity of these methods, comparisons between the regression models for ecological data with those for individual data need to be made. Because Rosenbaum and Rubin[4] have shown that the first approach cannot generate unbiased risk estimates and that the third approach can generate unbiased risk estimates, the focus of discussion is placed on the validity of the second approach.

When data on individuals are available, a regression model for predicting the risk of individuals can be expressed as the following:

(4) $$\text{Y}=\text{S}+{\text{B}}_{1}{\text{z}}_{1}+...+{\text{B}}_{5}{\text{z}}_{5}+\text{CU}+\text{Ft}+\varepsilon$$

where Y is an indicator for the outcome (for patients, Y = 1; otherwise, Y = 0), z*j *indicates exposures to arsenic (for people in exposure category *j*, z*j *= 1; otherwise, z*j *= 0), U is the urbanization index of the residential township, t is the number of cigarettes smoked, and ε is the error term. The number of cigarettes smoked by an individual was estimated by the number of cigarettes sold to that individual in the analyses. In this case, S indicates the background risk, B*j *indicates the additional risk associated with exposure to arsenic levels in category *j*, C indicates the additional risk associated with each one-unit increase in urbanization index, and F indicates the additional risk associated with each cigarette sold. Because Model 2 used relative risk as the dependent variable, it should be compared with the following model for individual data derived from Model 4:

(5) $$\begin{array}{c} \text{E}\left(\text{Y}∕\text{S}\right)=\text{S}∕\text{S}+{\text{B}}_{1}{\text{z}}_{1}∕\text{S}+...+{\text{B}}_{5}{\text{z}}_{5}∕\text{S}+\text{CU}∕\text{S}+\text{Ft}∕\text{S}+\varepsilon ∕\text{S}\\ \;\;\;\;=1+{\text{B}}_{1}^{\prime }{\text{z}}_{1}+...+{\text{B}}_{5}^{\prime }{\text{z}}_{5}+{\text{C}}^{\prime }\text{U}+{\text{F}}^{\prime }\text{t}+\varepsilon^{\prime } \end{array}$$

In this case, B*j*' indicates the additional relative risk associated with exposure to arsenic levels in category *j*, C' indicates the additional relative risk associated with each one-unit increase in urbanization index, and F' indicates the additional relative risk associated with each cigarette smoked (sold). However, an ecological study observed a township with n people would in fact observe the sum of n equations from Model 5:

$$\begin{array}{c} \text{N}∕\text{S}=\Sigma \left(1+{{\text{B}}_{1}}^{\prime }{\text{z}}_{1}i+...+{{\text{B}}_{5}}^{\prime }{\text{z}}_{5}i+{\text{ C}}^{\prime }\text{U}i+{\text{ F}}^{\prime }\text{t}i+\varepsilon^{\prime }i\right)\\ \;\;\;\;=\text{n}+{{\text{B}}_{1}}^{\prime }\Sigma {\text{z}}_{1}i+...+{{\text{B}}_{5}}^{\prime }\Sigma {\text{z}}_{5}i+{\text{C}}^{\prime }\Sigma \text{U}i+{\text{F}}^{\prime }\Sigma \text{t}i+\Sigma \varepsilon^{\prime }i, \end{array}$$

where N is the number of cases, and Σz*ji *is the number of residents in category *j*. Because every resident in a given township had the same urbanization index, ΣU*i *= nU. In addition, given the assumption that the errors (ε'*i*'s) are randomly distributed around 0 in each township, Σε'*i *has an expected value of 0. Therefore, the summary equation can be written as

$$\text{E}\left(\text{N}∕\text{S}\right)=\text{n}+{{\text{B}}_{1}}^{\prime }\Sigma {\text{z}}_{1}i+...+{{\text{B}}_{5}}^{\prime }\Sigma {\text{z}}_{5}i+{\text{C}}^{\prime }\text{nU}+{\text{F}}^{\prime }\Sigma \text{t}i.$$

When the dependent variable is the ratio of observed number of cases (N) to the expected number of cases (nS), the equation becomes

(6) $$\begin{array}{c} \text{E}\left(\text{N}∕\text{nS}\right)=\text{n}∕\text{n}+{{\text{B}}_{1}}^{\prime }\Sigma {\text{z}}_{1}i∕\text{n}+...+{{\text{B}}_{5}}^{\prime }\Sigma {\text{z}}_{5}i∕\text{n}+{\text{C}}^{\prime }\text{nU}∕\text{n}+{\text{F}}^{\prime }\Sigma \text{t}i∕\text{n}\\ \;\;\;\;=1+{{\text{B}}_{1}}^{\prime }{\text{X}}_{1}i∕100+...+{{\text{B}}_{5}}^{\prime }{\text{X}}_{5}i∕100+{\text{C}}^{\prime }\text{U}+100{\text{F}}^{\prime }\text{T}\\ \;\;\;\;=1+{{\text{B}}_{1}}^{\prime }∕100{\text{X}}_{1}i+...+{{\text{B}}_{5}}^{\prime }∕100{\text{X}}_{5}i+{\text{C}}^{\prime }\text{U}+100{\text{F}}^{\prime }\text{T} \end{array}$$

where, as defined in Model 2, X*j *denotes the percentage of residents in category *j*, and T is the average number of cigarettes (in hundreds) sold per capita. From Model 5 at the individual level to Model 6 at the township level, all regression coefficients (B*j*', C', and F') remain the same. Comparing Model 2 with Model 6, one can find that while the independent variables X*_j_*, U, and T are the same, SIRatio, which has been adjusted for age, is not identical or proportional to N/nS. Therefore βj', γ', and δ' are not unbiased estimates of Bj'/100, C', and 100F', and α' does not necessarily has an expected value of 1.

## Discussion

This study showed that different methods of age-adjustment may lead to different results and that the two methods (Direct and Indirect) frequently applied to adjust for age in ecological studies can not generate unbiased risk estimates, although the control of the effect of age may be achieved. Even though none of the risks associated with age groups was significant in this study, application of either method might lead to different conclusions, such as a significant positive association between urbanization index and bladder cancer women. On the other hand, the Variable Method not only adjusts for the effects of age, but also generates risk estimates associated with different age groups. Therefore, even though the Direct and Indirect Methods may generate valid age-adjusted risk estimates for individual unit population directly for the purposes of comparison among populations, one should not use these estimates as dependant variables for the purpose of age-adjustment when applying regression models to analyze ecological data.

When the effects of age themselves are not of primary interest, a reasonable alternative to conduct age-adjustment in ecological studies is to use the mean age of each unit population as an independent variable in the regression model. But, a study showed that this approach is based on the assumption of a linear dose-response relationship between age and the outcome of interest [5]. That study also showed that such assumptions are not necessary when one uses independent variables derived from dummy variables at the individual level in the regression models, as in the Variable Methods described above. When more data are available, other approaches may also be applied. For example, when we have age-specific data on both dependent and independent variables, we may conduct separate regression analyses for different age groups. However, such data are often unavailable, as in the case presented in this paper. We should also note that the transformation of Model 5 at the individual level to Model 6 at the township level only holds for linear regression, not for logistic or Poisson regressions, although they may be more appropriate for some cases.

The occurrence of urinary cancers has been noted among arsenic intoxicated patients in the 1950s [26], and high incidence of urinary cancers associated with arsenic in drinking water has reported in Taiwan [9,19] and many other countries [27-32]. Users of Fowler's solution (containing potassium arsenite) [33] and wine growers exposed to arsenical pesticides through drinking alcoholic beverages and spraying pesticides [34] had increased risks of bladder cancer. Therefore, the associations between arsenic ingestion and bladder cancer observed in this study are supported by the scientific literature. The data on the dose-response relationships, however, are quite limited. A study in Japan [29] observed cases only at the highest level among the three studied, and other studies in Taiwan also support the association between exposure to arsenic levels above 0.3 mg/L in drinking water and mortality of bladder cancer [14,35]. The irregularities in the dose-response relationships at lower exposure levels observed in this study among women might due to the effects of un-controlled confounders or the cell-types specificity of the carcinogenic effect of arsenic on the urinary system [9]. Therefore, the association between exposure to arsenic levels below 0.3 mg/L in drinking water and occurrence of bladder cancer needs further evaluation. In fact, a recent meta-analysis showed that arsenic levels below < 0.2 mg/L alone did not appear to be a significant independent risk factor for bladder cancer [36].

Absence of an association between cigarette sales and bladder cancer in this study is consistent with the findings of a previous study in Taiwan which showed associations between smoking and lung cancer, but not bladder cancer, after adjusting for exposures to arsenic [37]. The number of cigarettes sold in a township might not be a good surrogate measurement of the number of cigarettes actually consumed by the residents, and it is also possible that excess risks associated with smoking were too small to be detected by either study.

The ecologic study presented in this paper shares with all the other ecologic studies several major limitations inherent in the ecological study design, such as "ecological fallacy." Although it might be minimized by using smaller population units such as "village," this study had to use township as the unit because the National Cancer Registry Program coded the residences of cases by township. On the other hand, for a relatively rare disease like bladder cancer among Taiwanese, estimates of incidence rates will be unstable if the unit population is too small, especially when the duration of observation is relatively short. Risk estimates generated by this study might be affected by possible incomplete reporting of cancer cases because the reporting is not mandatory by law. If there is a correlation between reporting rates and arsenic exposure levels, proper validation studies on the registry, which are not currently available, are necessary to determine the direction and magnitude of the biases. Nonetheless, in a previous Taiwan study on skin cancer, which has a much more serious problem of under-reporting than bladder cancer because of its low case fatality rate, the comparison between the relative risks obtained by analyzing cancer registry data and those obtained by conducting physical examinations on all study participants to achieve complete case ascertainment showed that the possible under-reporting had little effect on the estimates of relative risks [38]. Except for the " < 0.05 mg/L" category (the reference exposure category), no township had all the wells in a single exposure category, and so it is impossible to validate the risks estimates in the current study. In addition, the current study cannot account for the migration of residents of each township over the 10-year study period. Further studies with exposure data for each individual (such as case-control or cohort studies) as well as studies evaluating the effects of other co-existing risk factors are needed to confirm the hypotheses generated in this study.

## Conclusions

Although ecological study design has some major limitations, it is widely applied in epidemiology. Like in other types of study designs, adjustment for age is often necessary in ecological studies, but different methods may lead to different conclusions. The current study compared three common approaches of age adjustment using a study on the associations between arsenic in drinking water and incidence of bladder cancer in 243 townships in Taiwan was used as an example. Whereas all three methods showed a statistically significant positive association between arsenic exposure above 0.64 mg/L and incidence of bladder cancer in both genders, they reached different conclusions on the other exposure categories. Even for the category above 0.64 mg/L, the risk estimates obtained by different methods were different. Using proportions of residents in different age groups as a part of the independent variables in the multiple regression models is the only approach of the three that can obtain unbiased estimates of the risks and also generate estimates of the risk associated with each age group. This approach is recommended for age adjustment in ecological studies.

## Abbreviations

CIR: crude cumulative incidence rate; IRR: incremental relative risk; RD: rate difference; SE: standard error; SIRate: standardized incidence rate; SIRatio: standardized incidence ratio.

## Competing interests

The author declares that he has no competing interests.

## Pre-publication history

The pre-publication history for this paper can be accessed here:

http://www.biomedcentral.com/1471-2458/11/820/prepub
